# Correction: Variation in surface properties, metabolic capping, and antibacterial activity of biosynthesized silver nanoparticles: comparison of bio-fabrication potential in phytohormone-regulated cell cultures and naturally grown plants

**DOI:** 10.1039/d0ra90116d

**Published:** 2020-11-09

**Authors:** Tariq Khan, Gul Shad Ali

**Affiliations:** Department of Biotechnology, University of Malakand Chakdara Dir Lower 18800 Pakistan tariqkhan@uom.edu.pk +92 3339546605; Plant Molecular and Cell Biology, Department of Plant Pathology, Mid-Florida Research and Education Center, University of Florida/Institute of Food and Agricultural Sciences Apopka FL USA gulali@eukaryotech.com; EukaryoTech LLC. Apopka FL 32703 USA +1 9706820484

## Abstract

Correction for ‘Variation in surface properties, metabolic capping, and antibacterial activity of biosynthesized silver nanoparticles: comparison of bio-fabrication potential in phytohormone-regulated cell cultures and naturally grown plants’ by Tariq Khan *et al.*, *RSC Adv.*, 2020, **10**, 38831–38840, DOI: 10.1039/D0RA08419K.

The authors regret that an incorrect version of Fig. 7 was included in the original article. The correct version of Fig. 7 is presented below.

**Fig. 7:**
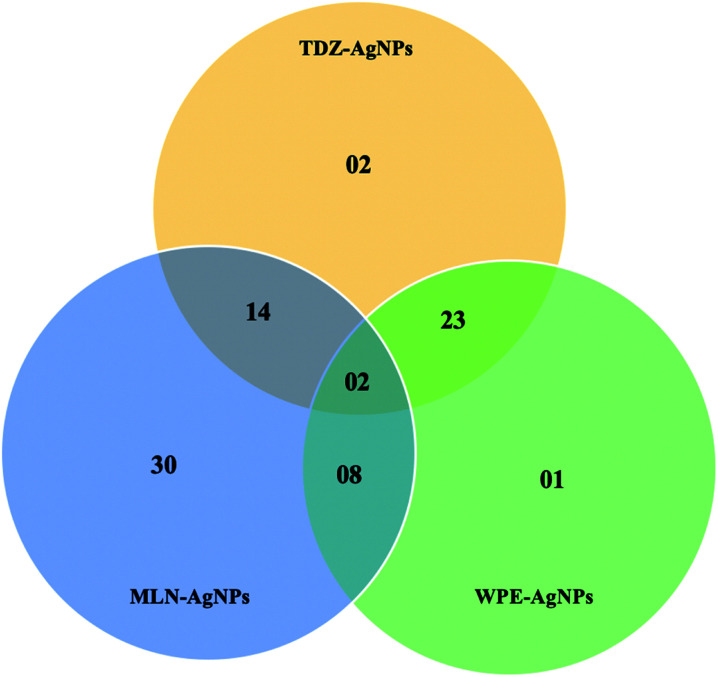
Venn diagram for the comparative analysis of compounds detected through LC-MS/MS.

The Royal Society of Chemistry apologises for these errors and any consequent inconvenience to authors and readers.

## Supplementary Material

